# Interval-valued bipolar fuzzy line graphs

**DOI:** 10.1186/s13104-023-06352-9

**Published:** 2023-06-26

**Authors:** Keneni Abera Tola, V. N. Srinivasa Rao Repalle, Mamo Abebe Ashebo

**Affiliations:** Department of Mathematics, Wollega University, Nekemte, Ethiopia

**Keywords:** Bipolar fuzzy graph, Interval-valued bipolar fuzzy line graph, Interval-valued bipolar fuzzy graph Isomorphism, Primary 05C72, Secondary 03B20

## Abstract

**Objectives:**

The notion of Bipolarity based on positive and negative outcomes. It is well known that bipolar models give more precision, flexibility, and compatibility to the system as compared to the classical models and fuzzy models. A bipolar fuzzy graph(BFG) provides more flexibility while modeling human thinking as compared with a fuzzy graph, and an interval valued bipolar fuzzy graph(IVBFG) has numerous applications where the real-life problem are time dependent and there is a network structure complexity. The aim of this paper is to introduce an interval-valued bipolar line fuzzy graph(IVBFLG).

**Result:**

In this paper, we have proposed the notion of an IVBFLG and some of its characterizations. Also, some propositions and theorems related to an IVIFLGs are developed and proved. Furthermore, isomorphism between two IVIFLGs toward their IVIFGs was determined and verified. As a result, we derive a necessary and sufficient condition for an IVBFG to be isomorphic to its corresponding IVBFLG and some remarkable properties like degree, size, order, regularity, strength, and completeness of an IVBFLGs have been investigated, and the proposed concepts are illustrated with the examples.

## Introduction

A graph structure is an appropriate method for solving combinatorial problems in computer science and soft computing systems. So that, researrhers By using classical graph The concept of bipolarity appears to pervade human decision making and understanding of explicit handling of positive and negative sides of information in the development of technology, which is very useful in cooperation and competition, friendship and hostility, common interests and conflicting interests, effect and side effect, likelihood and unlikelihood, feed forward and feedback [1].

In 1965, Zadeh replaced the classical set with a fuzzy set, which gives better exactness in both theory and application [2]. Afterwards, Kauffman proposed fuzzy graphs based on Zadeh’s fuzzy relations [3]. Later on, Rosenfeld [4] discussed the fuzzy analogue of many graph-theoretic concepts. Following this, researchers began to introduce many classes of fuzzy graphs, and they have made remarkable advances with impressive applications of fuzzy theory.

In 1994, Zhang [5] incorporated the idea of bipolar fuzzy sets as a generalization of fuzzy sets to overcome the double-sided thinking nature of humans in decision making. As explored in [6], a bipolar fuzzy set is an extension of Zadeh’s fuzzy set theory whose membership degree range is [− 1, 1]. In a bipolar fuzzy set, the membership degree of 0 of an element means that the element is irrelevant to the corresponding property, the membership degree (0, 1] of an element represents what is considered possible to the corresponding property, and the membership degree [− 1, 0) represents what is considered impossible or somewhat satisfies the implicit counter property corresponding to a BFS [7]. On the other hand, positive information describes what is possible, acceptable, permitted, wanted, or thought to be desirable, while negative information describes what is rejected, forbidden, or impossible. According to Bosc and Pivert, Bipolarity is the propensity of th e human mind to reason and make decisions on the basis of positive and negative effects [8]. This set is presented for cognitive modeling and multiagent decision analysis.

Bipolar fuzzy graphs have recently received a lot of attention from researchers. Akram and others presented the idea and the symbolization of the bipolar fuzzy graph (BFG), and also investigated the metric in bipolar fuzzy graphs, regular bipolar fuzzy graphs, irregular bipolar fuzzy graphs, antipodal bipolar fuzzy graphs, and bipolar fuzzy hypergraphs, as well as several properties with applications [9–14]. This notion was connected with the existence of bipolar information about the given set. The BFG can be used to model many problems in economics, operations research, etc. involving two similar, but opposite type of qualitative variables like success and failure, gain and loss [15]. A defined bipolar fuzzy graph was used to introduce the concept of a bipolar fuzzy line graph (BFLG). The structure of a line graph [L(G)] is typically more complex than that of the corresponding graph G. Likewise, to understand this complexity many other operations in graph theory were introduced and illustrated with examples [16–20]. In molecular graphs, topological indices are most important and have many useful applications. Some applications of these operations are presented in the field of Graph Theory. Amongst, the degree sequence of a graph gives many information about the properties of the topological indices and also the real life situations that the graph corresponds in various structural properties of graphs [21]. Particularly, problems that are difficult to solve on general graphs are frequently solved on line graphs. The line graph is obtained by associating a node with each edge and linking a node with an edge if the corresponding edges of the graph share a node. A large number of variants of line graphs like, classical line graphs [22], fuzzy line graphs [23], interval-valued fuzzy line graphs (IVFLG) [24], and the L(G) of interval valued intuitionistic fuzzy graph(IVIFG) [25] have been recently introduced in the literature. So far, an IVBFLG has not been studied. Some work on bipolar fuzzy graphs and notations not declared in this manuscript may be found on [27–34].

The primary contribution of this paper is as per the following:

- We introduce an interval-valued bipolar fuzzy line graph (IVBFLG).
- The brief introduction of bipolar fuzzy graphs (BFG) and related works were organized.
- Many propositions and theorems on the properties of IVBFLG are developed and proved.
- Further, interval-valued bipolar weak vertex homomorphism and interval-valued bipolar weak line isomorphism are proposed.

## Main text

In this paper, we considered only graphs without loops or multiple edges and undirected interval-valued bipolar fuzzy graphs.

### Definition 1

[26] An ordered triple $G=(~V,~ \sigma ,~ \mu )$ is said to be a fuzzy graph(FG) where $V=\{v_1, v_2,\cdots , v_n \}$ such that $\sigma :V\rightarrow [0,~1]$, and a fuzzy relation $\mu$ on $\sigma$ is $\mu : V\times V \rightarrow ~ [0,~1]$ satisfies that $\mu (u,v)\le ~ \sigma (u)\wedge \sigma (v),$ for all $u,v\in V.$

### Definition 2

Let X be a non-empty set. A bipolar fuzzy set A on X is an object having the form $A=\{( \text {x}, \mu _A^P(x),\mu _A^N(\text {x}))~:\text {x}\in X\}$ where $\mu _A^P(x):X\rightarrow [0,1]$ denotes a positive membership degree of the elements of X and $\mu _A^N(x):X\rightarrow [-1,0]$ denotes a negative membership degree of the elements of X.

### Definition 3

[27] For a nonempty set X, a mapping $B=(\sigma _B^P,\sigma _B^N ):X\times X\rightarrow [0,1]\times [-1,0]$ a bipolar fuzzy relation on X such that $\mu _B^P(x,y)\in [0,1]$ and $\mu _B^N(x,y)\in [-1,0]$.

### Definition 4

A bipolar fuzzy graph is defined to be a pair $G=(A,~B)$ where $A=(\sigma _A^P,\sigma _A^N )$ is a bipolar fuzzy set in a nonempty and finite set V and $B=(\sigma _B^P,\sigma _B^N )$ a bipolar fuzzy set on E satisfying $\sigma _B^P(v_iv_j)\le \sigma _A^P(v_i)\wedge \sigma _A^P(v_j)$ and $\sigma _B^N(v_iv_j)\ge \sigma _A^N(v_i)\vee \sigma _A^N(v_j) ~\forall ~ v_iv_j\in E.$

Here, we call A is a bipolar fuzzy vertex set of V and B is a bipolar fuzzy edge set of E.

### Definition 5

[28] Given a crisp graph $G^*$, its line graph $L(G^*)$ is a graph such that each vertex of $L(G^*)$ represents an edge of $G^*$, and two vertices of $L(G^*)$ are adjacent if and only if their corresponding edges share a common endpoint.

### Definition 6

Consider $L(G^*)=(Z, W)$ be line graph of $G^*=(V,E)$. Let $G=(A_1, B_1)$ be BFG of $G^*$. Then we define a bipolar fuzzy line graph $L(G) =(A_2,~B_2)$ of a bipolar fuzzy graph G as follows:

- $$\sigma _{A_2}^P(S_e) = \sigma _{B_1}^P(e)=\sigma _{B_1}^P(u_ev_e),$$ $$\sigma _{A_2}^N(S_e) = \sigma _{B_1}^N(e)=\sigma _{B_1}^N(u_ev_e),$$ for all $$S_e \in Z$$
- $$\sigma _{B_2}^P(S_eS_f) = \sigma _{B_1}^P(e)\wedge \sigma _{B_1}^P(f)$$ $$\sigma _{B_2}^N(S_eS_f) = \sigma _{B_1}^N(e)\wedge \sigma _{B_1}^N(f)$$, $$\forall ~ S_eS_f \in W.$$

where $A_2 = (\sigma _{A_2}^P, \sigma _{A_2}^N)$ is bipolar fuzzy sets of Z and $B_2 = (\sigma _{B_2}^P, \sigma _{B_2}^N)$ is bipolar fuzzy relation of W.

The line graph $L(G) = (A_2,B_2)$ of BFG G is always BFG.

### Definition 7

Suppose there are two BFG $G_1=(A_1, ~B_1)$ and $G_2=(A_2, ~B_2)$, then the mapping $\varphi : V_1 \rightarrow V_2$ is a homomorphism of $\varphi : G_1 \rightarrow G_2$ such that

- $$\sigma _{A_1}^P(v_i)\le \sigma _{A_2}^P(\varphi (v_i)), ~~~\sigma _{A_1}^N(v_i)\ge \sigma _{A_2}^N(\varphi (v_i))$$
- $$\sigma _{B_1}^P(v_i,v_j)\le \sigma _{B_2}^P(\varphi (v_i)\varphi (v_j)), ~~$$ $$\sigma _{B_1}^N(v_i,v_j)\ge \sigma _{B_2}^N(\psi (v_i)\varphi (v_j))$$ $$\forall ~v_i\in V_1, ~ v_iv_j\in E_1.$$

### Definition 8

For a non void universal set *X* and $A\subseteq X$, we define an interval-valued bipolar fuzzy set(IVBFS) of A as follows:

$$\begin{aligned}A=\{~([\lambda _A^{l}(v_i), \lambda _A^{u}(v_i)],[\mu _A^{l}(v_i),\mu _A^{u}(v_i) ]):~v_i\in X\}\end{aligned}$$

where, $$\lambda _A^{l} \le \lambda _A^{u}$$ and $$\mu _A^{l} \le \mu _A^{u}$$, $$\forall v_i \in V$$.

We use $\lambda _{~A~}^{l}(x),$ and $\lambda _{~A~}^{u}(x)$ to denote the lower and upper satisfaction degree of an element *x* respectively, to the property corresponding to a bipolar fuzzy set A, and also $\mu _{~A}^{l}(x),$ and $\mu _{~A}^{u}(x)$ represents the lower and upper satisfaction degree of an element *x* respectively, to some explicit or implicit property corresponding to a bipolar fuzzy set A.

### Definition 9

The graph $G=(A,B)$ is said to be IVBFG where $A~=(~ [\lambda _{~A~}^{l}(x), \lambda _{~A~}^{u}(x) ],$ $[ \mu _{~A~}^{l}(x), \mu _{~A~}^{u}(x)]),$ represent a IVBFS and $B~=(~ [\lambda _{~B~}^{l}(x), \lambda _{~B~}^{u}(x) ],$ $[ \mu _{~B~}^{l}(x), \mu _{~B~}^{u}(x)])$ is a IVBF- relation on $\lambda$, which satisfies the following conditions:

1. $$\lambda _B^l(v_iv_j)\le (\lambda _A^l(v_i)\wedge \lambda _A^l(v_j)$$ $$\lambda _B^u(v_iv_j)\le (\lambda _A^u(v_i)\wedge \lambda _A^u(v_j)$$
2. $$\mu _B^l(v_iv_j)\ge (\mu _A^l(v_i)\vee \mu _A^l(v_j)$$ $$\mu _B^u(v_iv_j)\ge (\mu _A^u(v_i)\vee \mu _A^u(v_j)$$ $$~~\forall ~v_iv_j \in E$$.

### Example 10

**Fig. 1:**
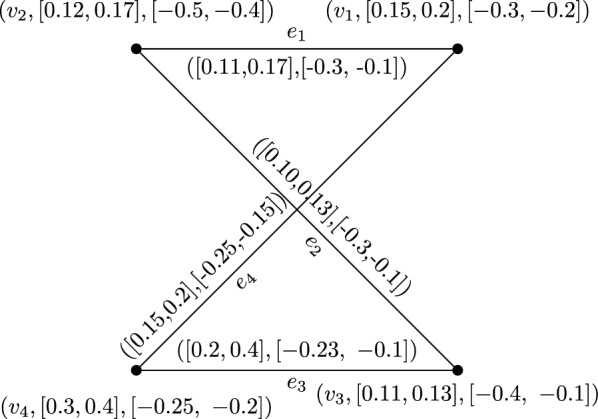
IVBFGG

Consider an interval valued bipolar fuzzy graph G from the below Fig. 1.

### Definition 11

An interval valued bipolar fuzzy graph $G =(A, B)$ is called strong if

$$\lambda _{B}^l(u,v)=\min \big (\lambda _{B}^l(u),\lambda _{B}^l(v)\big )$$ and $$\mu _{B}^l(u,v)=\max \big (\mu _{B}^l(u),\mu _{B}^l(v)\big )$$,

$\lambda _{B}^u(u,v)=\min \big (\lambda _{B}^u(u),\lambda _{B}^u(v)\big )$ and $\mu _{B}^u(u,v)=\max \big (\mu _{B}^u(u),\mu _{B}^u(v)\big )$, for all $(u,v)\in E$.

### Definition 12

[29] An interval valued bipolar fuzzy graph $G =(A, B)$ is called complete if

$$\lambda _{B}^l(u,v)=\min \big (\lambda _{B}^l(u),\lambda _{B}^l(v)\big )$$ and $$\mu _{B}^l(u,v)=\max \big (\mu _{B}^l(u),\mu _{B}^l(v)\big )$$,

$\lambda _{B}^u(u,v)=\min \big (\lambda _{B}^u(u),\lambda _{B}^u(v)\big )$ and $\mu _{B}^u(u,v)=\max \big (\mu _{B}^u(u),\mu _{B}^u(v)\big )$ for all $u,v\in V$.

The neighborhood of a vertex $\text {v} \in G$ is the induced subgraph of *G* consisting of all vertices adjacent to $\text {v}$ and all edges connecting two such vertices. Its often denoted by $\text {N(v)}$. The set of neighbors, known as a (open) neighborhood $N(\text {v})$ for a vertex $\text {v} \in G$, consists of all vertices adjacent to $\text {v}$ but not including $\text {v}$, i.e. $N(\text {v})=\{\text {u}\in V: \text {uv}\in E\}$. Equivalently, $deg(\text {v})=|N(\text {v})|$. When $\text {v}$ is also included, it is called a closed neighborhood, denoted $N[\text {v}]$ and $N[\text {v}]=N(\text {v})\cup \{\text {v}\}$.

### Definition 13

Let G be an interval-valued bipolar fuzzy graph. The neighborhood of a vertex $\text {v}$ in G is defined by $N(\text {v})=\big (N_{\lambda }(\text {v}),N_{\mu }(\text {v}) \big )$ where $N_{\lambda }(\text {v})=\{ [\lambda _B^l(\text {uv}), \lambda _B^u(\text {uv}] ~:~\lambda _B^l(\text {uv})\le \lambda _A^l(\text {u})\wedge \lambda _A^l(\text {v})~ \& ~ \lambda _B^u(\text {uv})\le \lambda _A^u(\text {u})\wedge \lambda _A^u(\text {v}) ~for ~\text {u}\in V,~ \text {uv}\in E\}$ and $N_{\mu }(\text {v})=\{ [\mu _B^l(\text {uv}), \mu _B^u(\text {uv}] ~:~\mu _B^l(\text {uv})\ge \lambda _A^l(\text {u})\vee \mu _A^l(\text {v})~ \& ~ \mu _B^u(\text {uv})\ge \mu _A^u(\text {u})\vee \mu _A^u(\text {v}) ~for ~\text {u}\in V,~ \text {uv}\in E\}$.

### Definition 14

The degree of a vertex $\text {v}\in V$ in a IVBFG G is denoted by $\deg (v)=\big (\deg \lambda (v),\deg \mu (v)\big )$ where $\deg \lambda (v)=[\deg \lambda ^l(v),\deg \lambda ^u(v)]$, $\deg \mu (v)= [\deg \mu ^l(v),\deg \mu ^u(v)]$ and defined as

$$\deg \lambda ^l(v)=\sum \limits _{ v\ne w }\lambda ^l_B(vw)$$,     $$\deg \lambda ^u(v)=\sum \limits _{v\ne w } \lambda ^u_B(vw),$$

$$\deg \mu ^l(v)=\sum \limits _{ v\ne w }\mu ^l_B(vw)$$,     $$\deg \mu ^u(v)=\sum \limits _{v\ne w } \mu ^u_B(vw),~\mathrm {~for~} vw\in E.$$

If $\deg (v)=(k_1, k_2)$, $\forall v\in V$ where $k_1=[k_1^l,k_1^u]$ and $k_2=[k_2^l,k_2^u]$, G is called $(k_1, k_2)$-regular.

The order of IVBFG, which is a pair of positive and negative orders of IVBFG, and the size of IVBFG, which is a pair of positive and negative sizes of IVBFG, are presented in the following definition.

### Definition 15

The order of a IVBFG G is denoted by $O(G)=(O_{\lambda }(G), O_{\mu }(G))$ where $O_{\lambda }(G)=[O^l_{\lambda }(G),O^u_{\lambda }(G) ]$ and $O_{\mu }(G)=[O^l_{\mu }(G),O^u_{\mu }(G) ]$ such that

$$\begin{aligned} O_{\lambda }(G)&=\big [O^l_{\lambda }(G), O^u_{\lambda }(G)\big ]\\&=\big [\sum \limits _{ v\in V } \lambda ^l_B(v),~\sum \limits _{ v\in V } \lambda ^u_B(v)\big ], \\ O_{\mu }(G)&=\big [O^l_{\mu }(G), O^u_{\mu }(G)\big ]\\&=\big [\sum \limits _{ v\in V } \mu ^l_B(v),~\sum \limits _{ v\in V } \mu ^u_B(v)\big ]. \end{aligned}$$

Also, $S(G)=(S\lambda (G),S\mu (G))$ is the size of G, where

$$\begin{aligned} S_{\lambda }(G)&=\big [S^l_{\lambda }(G), S^u_{\lambda }(G)\big ]\\&=\big [\sum \limits _{ vw\in E } \lambda ^l_B(vw),~\sum \limits _{ vw\in E } \lambda ^u_B(vw)\big ], \\ S_{\mu }(G)&=\big [S^l_{\mu }(G), S^u_{\mu }(G)\big ]\\&=\big [\sum \limits _{ vw\in E } \mu ^l_B(vw),~\sum \limits _{ vw\in E } \mu ^u_B(vw)\big ]. \end{aligned}$$

### Definition 16

The degree of an edge $vw\in E$ in a IVBFG G is denoted by $\deg (vw)=(deg\lambda (vw), deg\mu (vw))$ and is defined as

$$\begin{aligned} \deg \lambda (vw) =\, & [\deg {\lambda ^l}(vw),\;\deg {\lambda ^u}(vw)] \\ =\, & [\sum\limits_{vy \in E,} {\lambda _B^l} (vy) + \sum\limits_{wy \in E} {\lambda _B^l} (wy),\sum\limits_{vy \in E} {\lambda _B^u} (vy) + \sum\limits_{wy \in E} {\lambda _B^u} (wy)] \\ =\, & [\deg {\lambda ^l}(v) + \deg {\lambda ^l}(w) - 2\lambda _B^l(vw),\deg {\lambda ^u}(v) + \deg {\lambda ^u}(w) - 2\lambda _B^u(vw)] \\ \end{aligned}$$

$$\begin{aligned} \deg \mu (vw) & = [\deg {\mu ^l}(vw),\;\deg {\mu ^u}(vw)] \\ & = [\sum\limits_{vy \in E,} {\mu _B^l} (vy) + \sum\limits_{wy \in E} {\mu _B^l} (wy),\sum\limits_{vy \in E} {\mu _B^u} (vy) + \sum\limits_{wy \in E} {\mu _B^u} (wy)] \\ & = [\deg {\mu ^l}(v) + \deg {\mu ^l}(w) - 2\mu _B^l(vw),\deg {\mu ^u}(v) + \deg {\mu ^u}(w) - 2\mu _B^u(vw)]\, \\ & {\text{where}}\;\;y \ne v\;{\text{and}}\;y \ne w. \\ \end{aligned}$$

If $\deg (vw)=(r_1, r_2)$, $\forall vw\in E$ where $r_1=[r_1^l,r_1^u]$ and $r_2=[r_2^l,r_2^u]$, G is called $(r_1, r_2)$-edge regular.

### Example 17

By usual calculations degree of edge $e_1=v_1v_2$ is $\deg (e_1)=[\deg \lambda (e_1),\deg \mu (e_1)]$ in IVBFG G shown in Fig. 1.

$$\begin{aligned} \deg \lambda ({e_1}) =\, & [\deg {\lambda ^l}({e_1}),\;\deg {\lambda ^u}({e_1})] \\ =\, & [\deg {\lambda ^l}({v_1}{v_2}),\;\deg {\lambda ^u}({v_1}{v_2})] \\ =\, & [\deg {\lambda ^l}({v_1}) + \deg {\lambda ^l}({v_2}) - 2\lambda _B^l({v_1}{v_2}),\deg {\lambda ^u}({v_1}) + \deg {\lambda ^u}({v_2}) - 2\lambda _B^u({v_1}{v_2})] \\ =\, & [0.26 + 0.21 - 2(0.11),0.37 + 0.30 - 2(17)] \\ =\, & [0.47 - 0.22,0.67 - 0.34] \\ =\, & [0.25,0.33]. \\ \end{aligned}$$

$$\begin{aligned} \deg \mu ({e_1}) =\, & [\deg {\mu ^l}({e_1}),\;\deg {\mu ^u}({e_1})] \\ =\, & [\deg {\mu ^l}({v_1}{v_2}),\;\deg {\mu ^u}({v_1}{v_2})] \\ =\, & [\deg {\mu ^l}({v_1}) + \deg {\mu ^l}({v_2}) - 2\mu _B^l({v_1}{v_2}),\deg {\mu ^u}({v_1}) + \deg {\mu ^u}({v_2}) - 2\mu _B^u({v_1}{v_2})] \\ =\, & [ - 0.55 + ( - 0.6) - 2( - 0.3), - 0.25 + ( - 0.20) - 2( - 0.1)] \\ =\, & [ - 0.61 + 0.60, - 0.45 + 0.20] \\ =\, & [ - 0.1, - 0.25] \\ \end{aligned}$$

### Definition 18

The closed neighborhood degree (CND) of a vertex $v \in V$ in an IVBFG G is denoted by $\deg [v] = (\big [\deg \lambda ^l[v],~ \deg \lambda ^u[v]\big ],\big [\deg \lambda ^l[v], ~\deg \lambda ^u[v]\big ])$ and is defined as

$$\begin{array}{*{20}{c}} {\deg {\lambda ^l}[v] = \deg {\lambda ^l}(v) + \lambda _A^l(v),}&{\deg {\lambda ^u}[v] = \deg {\lambda ^u}(v) + \lambda _A^u(v)} \\ {\deg {\mu ^l}[v] = \deg {\mu ^l}(v) + \mu _A^l(v),}&{\deg {\mu ^u}[v] = \deg {\mu ^u}(v) + \mu _A^u(v).} \end{array}$$

If $\deg [v] = ( f_1, f_2) ~\forall v \in V,$ then G is called $( f_1, f_2)$-totally regular, where $A = \big ([\lambda ^l_A,\lambda ^u_A], [\mu ^l_A,\mu ^u_A]\big )$ and $B = \big ([\lambda ^l_B,\lambda ^u_B], [\mu ^l_B,\mu ^u_B]\big )$ are an interval valued bipolar fuzzy sets in V and E, respectively. The minimum degree and maximum degree of IVBFG G are $\sigma _E(G)=\wedge \{\deg _G(uv), \forall ~uv \in E\}$ and $\Delta _E(G)=\vee \{\deg _G(uv), \forall ~uv \in E\}$.

### Definition 19

Let $G = (A, B)$ be an IVBFG. Then G is said to be effective fuzzy graph if $\lambda ^l_B(uv) = \lambda ^l_A (u) \wedge \lambda ^l_A(v), \lambda ^l_B(uv) = \lambda ^u_A (u) \wedge \lambda ^u_A(v), \mu ^l_B(uv) = \mu ^l_A (u) \vee \mu ^l_A(v)$ and $\mu ^l_B(uv) = \mu ^l_A (u) \vee \mu ^l_A(v)$ for all $uv\in V\times V$.

### Definition 20

An interval valued bipolar fuzzy graph *G* is connected if any two vertices are joined by a path.

### Definition 21

An IVBFG $G= (A, B)$ is called strongly regular if the following axioms are satisfied:-

i) G is a k-regular IVBFG,
ii) The sum of the membership values of the vertices common to the adjacent vertices is the same for all adjacent pairs of vertices,
iii) The sum of the membership values of the vertices common to the non-adjacent vertices is the same for all non-adjacent pairs of vertices.

### Definition 22

Consider an intersection graph $P(S) = (S, T)$ of a crisp graph $G^*= (V, E)$. Let $A_1 = (\lambda _{A_1}, \mu _{A_1})$ and $B_1 = (\lambda _{B_1}, \mu _{B_1})$ be an interval-valued bipolar fuzzy sets on V and E, $A_2 = (\lambda _{A_2}, \mu _{A_2})$ and $B_2 = (\lambda _{B_2}, \mu _{B_2})$ on S and T, respectively. Then an interval-valued bipolar fuzzy intersection graph of the interval-valued bipolar fuzzy graph $G = (A_1, B_1)$ is an interval-valued bipolar fuzzy graph $P(G) = (A_2, B_2)$ such that

- $$\lambda _{A_2}(S_i)=[\lambda _{A_2}^l(S_i),~\lambda _{A_2}^u(S_i)]=[\lambda _{A_1}^l(v_i),~\lambda _{A_1}^u(v_i)]$$ $$\mu _{A_2}(S_i)=[\mu _{A_2}^l(S_i),~\mu _{A_2}^u(S_i)]=[\mu _{A_1}^l(v_i),~\mu _{A_1}^u(v_i)]$$
- $$\lambda _{B_2}(S_iS_j)=[\lambda _{B_2}^l(S_iS_j),~\lambda _{B_2}^u(S_iS_j)]=[\lambda _{B_1}^l(v_iv_j),~\lambda _{B_1}^u(v_iv_j)]$$ $$\mu _{B_2}(S_iS_j)=[\mu _{B_2}^l(S_iS_j),~\mu _{B_2}^u(S_iS_j)]=[\mu _{B_1}^l(v_iv_j),~\mu _{B_1}^u(v_iv_j)]$$

for every $$S_i, Sj\in S, S_iS_j\in T.$$

### Definition 23

Let $L(G^*)=(Z,~W)$ be a line graph of a crisp graph $G^*= (V, E).$ and $G=(A_1,B_1)$ be IVBFG, we define an interval valued bipolar fuzzy line graphs $L(G)=(A_2,B_2)$ whose functions of membership value is defined as

i) $A_2$ is IVBFS of Z and $B_2$ is IVBF-relation of W, such that $\lambda _{A_2}^l(S_e)=\lambda _{B_1}^l(e) = \lambda _{B_1}^l(u_ev_e)$ $\lambda _{A_2}^u(S_e)=\lambda _{B_1}^u(e) = \lambda _{B_1}^u(u_ev_e)$ $\mu _{A_2}^l(S_e)=\mu _{B_1}^l(e) = \mu _{B_1}^l(u_ev_e)$ $\mu _{A_2}^u(S_e)=\mu _{B_1}^u(e) = \mu _{B_1}^u(u_ev_e), ~~\forall ~S_e\in Z.$
ii) The edge set of L(G) is $\lambda _{B_2}^l(S_{e}S_{f})= \min (\lambda _{B_1}^l(e),~ \lambda _{B_1}^l(f)), ~~~~~~~$ $\lambda _{B_2}^u(S_{e}S_{f})= \min (\lambda _{B_1}^u(e),~ \lambda _{B_1}^u(f))$ $\mu _{B_2}^l(S_{e}S_{f})= \max (\mu _{B_1}^l(e),~ \mu _{B_1}^l(f)), ~~~~~~~$ $\mu _{B_2}^u(S_{e}S_{f})= \max (\mu _{B_2}^u(e),~ \mu _{B_1}^u(f) )$, for all $\, S_eS_f \in W.$

where $L(G^*)=(Z,W)$ be line graph of a crisp graph $G^*=(V,~E)$.

### Example 24

From IVBFG *G* of shown in Fig. 1, we can drive a IVBFLG as follow.

$$\begin{aligned} \lambda _{A_2} (S_{e_1})=\, & {} [\lambda _{B_1}^l (e_1),\lambda _{B_1}^u (e_1)]=\,[0.11, 0.17]\\ \lambda _{A_2}(S_{e_2})=\, & {} [\lambda _{B_1}^l (e_2),\lambda _{B_1}^u (e_2)]=\,[0.10,0.13]\\ \lambda _{A_2} (S_{e_3})=\, & {} [\lambda _{B_1}^l (e_3),\lambda _{B_1}^u (e_3)]=\,[0.2,0.4]\\ \lambda _{A_2}(S_{e_4})=\, & {} [\lambda _{B_1}^l (e_4),\lambda _{B_1}^u (e_4)]=\,[0.15,0.2]\\ \mu _{A_2} (S_{e_1})=\, & {} [\mu _{B_1}^l (e_1),\mu _{B_1}^u (e_1)]=\,[-0.3,~-0.1]\\ \mu _{A_2}(S_{e_2})=\, & {} [\mu _{B_1}^l (e_2),\mu _{B_1}^u (e_2)]=\,[-0.3,-0.1]\\ \mu _{A_2} (S_{e_3})=\, & {} [\mu _{B_1}^l (e_3),\mu _{B_1}^u (e_3)]=\,[-0.23,~-0.1]\\ \mu _{A_2}(S_{e_4})=\, & {} [\mu _{B_1}^l (e_4),\mu _{B_1}^u (e_4)]=\,[-0.25,-0.15]\\ \\ \lambda _{B_2} (S_{e_1} S_{e_2})=\, & {} [\min (\lambda _{B_1}^l(e_1),~ \lambda _{B_1}^l(e_2)), \min ( \lambda _{B_1}^u(e_1),~ \lambda _{B_1}^u(e_2))]=\,[0.10,0.13]\\ \lambda _{B_2} (S_{e_2} S_{e_3})=\, & {} [\min (\lambda _{B_1}^l(e_2),~ \lambda _{B_1}^l(e_3)), \min ( \lambda _{B_1}^u(e_2),~ \lambda _{B_1}^u(e_3))]=\,[0.10,0.13]\\ \lambda _{B_2} (S_{e_3} S_{e_4})=\, & {} [\min (\lambda _{B_1}^l(e_3),~ \lambda _{B_1}^l(e_4)), \min (\lambda _{B_1}^u(e_3),~ \lambda _{B_1}^u(e_4))]=\,[0.15,0.20]\\ \lambda _{B_2} (S_{e_2} S_{e_3})=\, & {} [\min (\lambda _{B_1}^l(e_4),~ \lambda _{B_1}^l(e_1)), \min (\lambda _{B_1}^u(e_4),~ \lambda _{B_1}^u(e_1))]=\,[0.11,0.17]\\ \\ \mu _{B_2} (S_{e_1} S_{e_2})=\, & {} [\max (\mu _{~B_1}^l(e_1), \mu _{~B_1}^l(e_2)), ~ \max (\mu _{~B_1}^u(e_1),~ \mu _{~B_1}^u(e_2))]=\,[-0.3,-0.1]\\ \mu _{B_2} (S_{e_2} S_{e_3})=\, & {} [\max (\mu _{~B_1}^l(e_2),~ \mu _{~B_1}^l(e_3)), \max (\mu _{~B_1}^u(e_2),~ \mu _{~B_1}^u(e_3))]=\,[-0.23,-0.10]\\ \mu _{B_2} (S_{e_3} S_{e_4})=\, & {} [\max (\mu _{~B_1}^l(e_3),~ \mu _{~B_1}^l(e_4)), \max ( \mu _{~B_1}^u(e_3),~ \mu _{~B_1}^u(e_4))]=\,[-0.3, -0.10]\\ \mu _{B_2} (S_{4} S_{e_1})=\, & {} [\max (\mu _{~B_1}^l(e_4),~ \mu _{~B_1}^l(e_1)), \max (\mu _{~B_1}^u(e_4),~ \mu _{~B_1}^u(e_1))]=\,[ -0.25,-0.10].\\ \end{aligned}$$

**Fig. 2:**
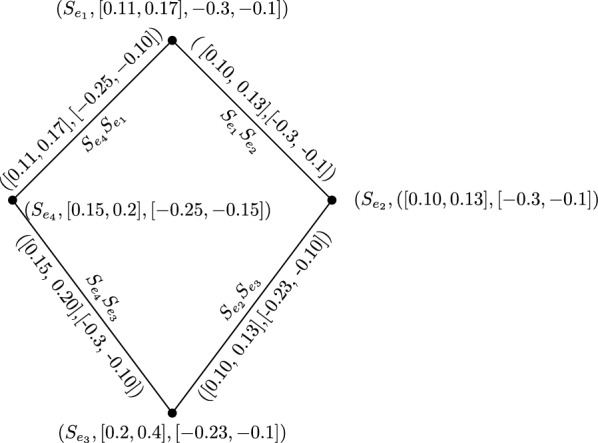
IVBFLG of G

Then L(G) of IVBFG G is shown in Fig. 2.

### Definition 25

Let $G_1 = (M_1, N_1)$ and $G_2 = (M_2, N_2)$ be IVBFGs. Then $\psi : G_1\rightarrow G_2$ is called homomorphism map if the following conditions are satisfied:

i) $$\lambda _{ M_1}^l( v_i)\le \lambda _{ M_2}^l( \psi (v_i))$$ $$\lambda _{ M_1}^u (v_i)\le \lambda _{ M_2}^u( \psi (v_i))$$ $$\mu _{ M_1}^l (v_i)\ge \mu _{ M_2}^l( \psi (v_i))$$ $$\mu _{ M_1}^u (v_i)\ge \mu _{ M_2}^u( \psi (v_i))$$, for every $$v_i\in V_1.$$
ii) $\lambda _{ N_1}^l (v_iv_j)\le \lambda _{ N_2}^l( \psi (v_i) \psi (v_j))$ $\lambda _{ N_1}^u (v_iv_j)\le \lambda _{ N_2}^u( \psi (v_i) \psi (v_j))$ $\mu _{ N_1}^l (v_iv_j)\ge \mu _{ N_2}^l( \psi (v_i) \psi (v_j))$ $\mu _{ N_1}^u (v_iv_j)\ge \mu _{ N_2}^u( \psi (v_i) \psi (v_j))$, for every $v_iv_j\in E_1$.

### Definition 26

An isomorphism between $G_1 = (M_1, N_1)$ and $G_2 = (M_2, N_2)$ is a bijective mapping $\psi :G_1\rightarrow G_2$ is called isomorphism if $\psi :V_1\rightarrow V_2$ such that,

i) $$\lambda _{ M_1}^l(v)= \lambda _{ M_2}^l( \psi (v))$$ $$\lambda _{ M_1}^u (v)= \lambda _{ M_2}^u( \psi (v))$$ $$\mu _{ M_1}^l (v)= \mu _{ M_2}^l( \psi (v))$$ $$\mu _{ M_1}^u (v)= \mu _{ M_2}^u( \psi (v))$$, for all $$v\in V_1.$$
ii) $\lambda _{ N_1}^l (v_iv_j)= \lambda _{ N_2}^l( \psi (v_i) \psi (v_j))$ $\lambda _{ N_1}^u (v_iv_j)= \lambda _{ N_2}^u( \psi (v_i) \psi (v_j))$ $\mu _{ N_1}^l (v_iv_j)= \mu _{ N_2}^l( \psi (v_i) \psi (v_j))$ $\mu _{ N_1}^u (v_iv_j)= \mu _{ N_2}^u( \psi (v_i) \psi (v_j))$ for all $v_iv_j\in E_1$.

### Definition 27

A weak vertex isomorphism between $G_1 = (M_1, N_1)$ and $G_2 = (M_2, N_2)$ is bijective mapping $\psi :V_1 \rightarrow V_2$ such that

1. $$\lambda _{M_1} (v_i)=\lambda _{ M_2} (\psi (v_i))$$ which means $$[\lambda _{ M_1}^l (v_i),\lambda _{ M_1}^u (v_i)]= [\lambda _{ M_2}^l (\psi (v_i)),\lambda _{ M_2}^u (\psi (v_i))],$$
2. $$\mu _{ N_1} (v_i)= \mu _{ N_2} (\psi (v_i))$$ which means $$[ \mu _{ N_1}^l (v_i), \mu _{ N_1}^u (v_i)]= [ \mu _{ N_2}^l (\psi (v_i)), \mu _{ N_2}^u (\psi (v_i))]$$,$$\forall v_i\in V_1.$$

This preserves only weight of the vertices not necessary weight of an edges. And also, $\psi :G_1 \rightarrow G_2$ is said to be a weak line isomorphism if

3. $$\lambda _{N_1} (v_iv_j)=\lambda _{N_2}( \psi (v_i) \psi (v_j))$$ which implies $$[\lambda _{N_1}^l (v_iv_j),~ \lambda _{N_1}^u(v_iv_j)]= [\lambda _{N_2}^l( \psi (v_i) \psi (v_j)), ~\lambda _{N_2}^u( \psi (v_i) \psi (v_j))]$$,
4. $$\mu _{N_1} (v_iv_j)= \mu _{N_2}^l( \psi (v_i) \psi (v_j))$$ which implies $$[ \mu _{N_1}^l (v_iv_j),~ \mu _{N_1}^u(v_iv_j)]= [ \mu _{N_2}^l( \psi (v_i) \psi (v_j)), ~ \mu _{N_2 }^u( \psi (v_i) \psi (v_j))],$$ $$~ \forall v_iv_j\in E_1.$$

This preserves only weight of the edges not necessary weight of a vertices.

### Example 28

Let $G_1 = (M_1, N_1)$ and $G_2 = (M_2, N_2)$ be IVBFGs.

**Fig. 3:**
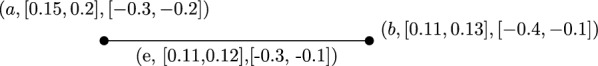
IVBFG $$G_1$$

**Fig. 4:**
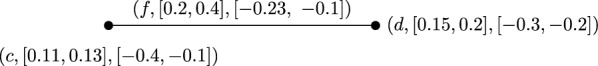
IVBFG $$G_2$$

Consider $\psi :V_1 \rightarrow V_2$ is the mapping from IVBFG $G_1$ into $G_2$. From Figs. 3 and 4, we have

$$\begin{aligned}\psi (a)=d, ~\psi (b)=c\end{aligned}$$

For all $\text {v}\in V_1$. So that its weak vertex isomorphism. But, its not weak line isomorphism since

$$\begin{aligned}\lambda _{N_1} (ab)\ne \lambda _{N_2}( \psi (a) \psi (b)) ~\mathrm {and~} \mu _{N_1} (ab)\ne \lambda _{N_2}( \psi (a) \psi (b)) \end{aligned}$$

### Definition 29

If the mapping $\psi : G_1 \rightarrow G_2$ is bijective weak vertex and weak edge isomorphism, then we said that $\psi$ is weak isomorphism map of IVBFG.

### Definition 30

Let $L(G)=(A_L,B_L)$ be an IVBFLG, then degree of a vertex $S_x\in V(L(G))$ in a graph G is denoted by $deg(S_x)=\big ([deg\lambda ^l(S_x),deg\lambda ^u(S_x)], [deg\mu ^l(S_x),deg\mu ^u(S_x)]\big )$ and is defined as

$$\begin{aligned} \deg {\lambda ^l}({S_x}) = & \sum\limits_{{S_x} \ne {S_y}} {\lambda _{{B_L}}^l} ({S_x}{S_y})\deg {\lambda ^u}({S_x}) \\ = & \sum\limits_{{S_x} \ne {S_y}} {\lambda _{{B_L}}^u} ({S_x}{S_y})\deg {\mu ^l}({S_x}) \\ = & \sum\limits_{{S_x} \ne {S_y}} {\mu _{{B_L}}^l} ({S_x}{S_y})\deg {\mu ^u}({S_x}) \\ = & \sum\limits_{{S_x} \ne {S_y}} {\mu _{{B_L}}^u} ({S_x}{S_y}),\;\;{\text{for}}\;{S_x}{S_y} \in E(L(G)). \\ \end{aligned}$$

If $\deg (S_x)=(k_1, k_2)$, $\forall S_x\in V(L(G))$ where $k_1=[k_1^l,k_1^u]$ and $k_2=[k_2^l,k_2^u]$, L(G) is said to be $(k_1, k_2)$- vertex regular interval-valued bipolar fuzzy line graph.

### Definition 31

The order of an IVBFLG G is denoted by $O(L(G))=(O_{\lambda }(L(G)), O_{\mu }(L(G)))$ where $O_{\lambda }(L(G))=[O^l_{\lambda }(L(G)),O^u_{\lambda }(L(G)) ]$ and $O_{\mu }(L(G))=[O^l_{\mu }(L(G)),O^u_{\mu }(L(G)) ]$ such that

$$\begin{aligned} O_{\lambda }(L(G))&=\big [O^l_{\lambda }(L(G)), O^u_{\lambda }(L(G))\big ]\\&=\big [\sum \limits _{ S_x\in V } \lambda ^l_{B_L}(S_x),~\sum \limits _{ S_x\in V } \lambda ^u_{B_L}(S_x)\big ] \\ O_{\mu }(L(G))&=\big [O^l_{\mu }(L(G)), O^u_{\mu }(L(G))\big ]\\&=\big [\sum \limits _{ S_x\in V } \mu ^l_{B_L}(S_x),~\sum \limits _{ S_x\in V } \mu ^u_{B_L}(S_x)\big ]. \end{aligned}$$

Also, $S(L(G))=(S\lambda (L(G)),S\mu (L(G)))$ is the size of G, where

$$\begin{aligned} S_{\lambda }(L(G))&=\big [S^l_{\lambda }(L(G)), S^u_{\lambda }(L(G))\big ]\\&=\big [\sum \limits _{ S_xS_y\in E } \lambda ^l_{B_L}(S_xS_y),~\sum \limits _{ S_xS_y\in E } \lambda ^u_{B_L}(S_xS_y)\big ] \\ S_{\mu }(L(G))&=\big [S^l_{\mu }(L(G)), S^u_{\mu }(L(G))\big ]\\&=\big [\sum \limits _{ S_xS_y\in E } \mu ^l_{B_L}(S_xS_y),~\sum \limits _{ S_xS_y\in E } \mu ^u_{B_L}(S_xS_y)\big ]. \end{aligned}$$

### Definition 32

The degree of an edge $S_xS_y\in E$ in an IVBFLG G is denoted by $deg (S_xS_y)=(\deg \lambda (S_xS_y), \deg \mu (S_xS_y))$ and is defined as

$$\begin{aligned} \deg \lambda ({S_x}{S_y}) = {\mkern 1mu} & \,[\deg {\lambda ^l}({S_x}{S_y}),\;\deg {\lambda ^u}({S_x}{S_y})] \\ = & {\mkern 1mu} \,\left[ {\sum\limits_{{S_x}{S_w} \in E,} {\lambda _{{B_L}}^l} ({S_x}{S_w}) + \sum\limits_{{S_y}{S_w} \in E} {\lambda _{{B_L}}^l} ({S_y}{S_w}),\sum\limits_{{S_x}{S_w} \in E} {\lambda _{{B_L}}^u} ({S_x}{S_w}) + \sum\limits_{{S_y}{S_w} \in E} {\lambda _{{B_L}}^u} ({S_y}{S_w})} \right] \\ = & \,\left[ {\deg {\lambda ^l}({S_x}) + \deg {\lambda ^l}({S_y}) - 2\lambda _{{B_L}}^l({S_x}{S_y}),\deg {\lambda ^u}({S_x}) + \deg {\lambda ^u}({S_y}) - 2\lambda _{{B_L}}^u({S_x}{S_y})} \right] \\ \end{aligned}$$

$$\begin{aligned} \deg \mu ({S_x}{S_y}) = {\mkern 1mu} & \,\,\left[ {\deg {\mu ^l}({S_x}{S_y}),\;\deg {\mu ^u}({S_x}{S_y})} \right] \\ = & {\mkern 1mu} \,\left[ {\sum\limits_{{S_x}{S_w} \in E,} {\mu _{{B_L}}^l} ({S_x}{S_w}) + \sum\limits_{{S_y}{S_w} \in E} {\mu _{{B_L}}^l} ({S_y}{S_w}),\sum\limits_{{S_x}{S_w} \in E} {\mu _{{B_L}}^u} ({S_x}{S_w}) + \sum\limits_{{S_y}{S_w} \in E} {\mu _{{B_L}}^u} ({S_y}{S_w})} \right] \\ = & \,{\mkern 1mu} \left[ {\deg {\mu ^l}({S_x}) + \deg {\mu ^l}({S_y}) - 2\mu _{{B_L}}^l({S_x}{S_y}),\deg {\mu ^u}({S_x}) + \deg {\mu ^u}({S_y}) - 2\mu _{{B_L}}^u({S_x}{S_y})} \right]. \\ \end{aligned}$$

If $\deg (S_xS_y)=(r_1, r_2)$, $\forall S_xS_y\in E$ where $r_1=[r_1^l,r_1^u]$ and $r_2=[r_2^l,r_2^u]$, L(G) is said to be $(r_1, r_2)$-edge regular IVBFLG.

### Definition 33

The closed neighborhood degree of a vertex $S_x \in V$ in an IVBFLG G is denoted by $\deg [S_x] = (\big [\deg \lambda ^l[S_x],~ \deg \lambda ^u[S_x]\big ],\big [\deg \lambda ^l[S_x], ~\deg \lambda ^u[S_x]\big ])$ and is defined as

$$\begin{array}{*{20}{c}} {\deg {\lambda ^l}[{S_x}] = \deg {\lambda ^l}({S_x}) + \lambda _{{A_L}}^l({S_x}),}&{\deg {\lambda ^u}[{S_x}] = \deg {\lambda ^u}({S_x}) + \lambda _{{A_L}}^u({S_x})} \\ {\deg {\mu ^l}[{S_x}] = \deg {\mu ^l}({S_x}) + \mu _{{A_L}}^l({S_x}),}&{\deg {\mu ^u}[{S_x}] = \deg {\mu ^u}({S_x}) + \mu _{{A_L}}^u({S_x}).{\text{ }}} \end{array}$$

If $\deg [S_x] = ( f_1, f_2) ~\forall S_x \in V,$ then L(G) is called $( f_1, f_2)$-totally regular, where $A_L = \big ([\lambda ^l_{A_L},\lambda ^u_{A_L}], [\mu ^l_{A_L},\mu ^u_{A_L}]\big )$ and $B_L = \big ([\lambda ^l_{B_L},\lambda ^u_{B_L}], [\mu ^l_{B_L},\mu ^u_{B_L}]\big )$ are an interval valued bipolar fuzzy sets in V and E, respectively. The minimum degree and maximum degree of IVBFLG G are $\sigma _E(L(G))=\wedge \{\deg _{L(G)}(uv), \forall ~S_xS_y \in E\}$ and $\Delta _E(L(G))=\vee \{\deg _{L(G)}(uv), \forall ~S_xS_y \in E\}$, respectively.

### Definition 34

An IVBFLG $L(G) =(A_L, B_L)$ is strong IVBFLG if and only if all of the following are holds

$$\lambda _{B_L}^l(s_es_f)=\min \big (\lambda _{B_L}^l(s_e),\lambda _{B_L}^l(s_f)\big )$$ and $$\mu _{B_L}^l(s_e,s_f)=\max \big (\mu _{B_L}^l(s_e),\mu _{B_L}^l(s_f)\big )$$,

$\lambda _{B_L}^u(s_e,s_f)=\min \big (\lambda _{B_L}^u(s_e),\lambda _{B_L}^u(s_f)\big )$ and $\mu _{B_L}^u(s_e,s_f)=\max \big (\mu _{B_L}^u(s_e),\mu _{B_L}^u(s_f)\big )$ for all $S_eS_f \in E(L(G))$.

**Remark:** If G is a regular IVBFG then *L*(*G*) need not be regular.

### Example 35

**Fig. 5:**
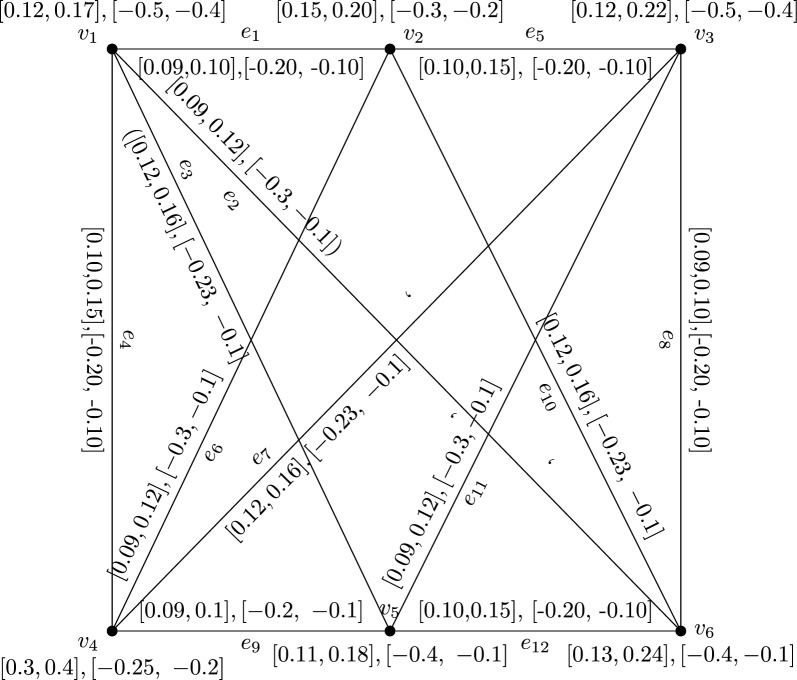
IVBFG H

**Fig. 6:**
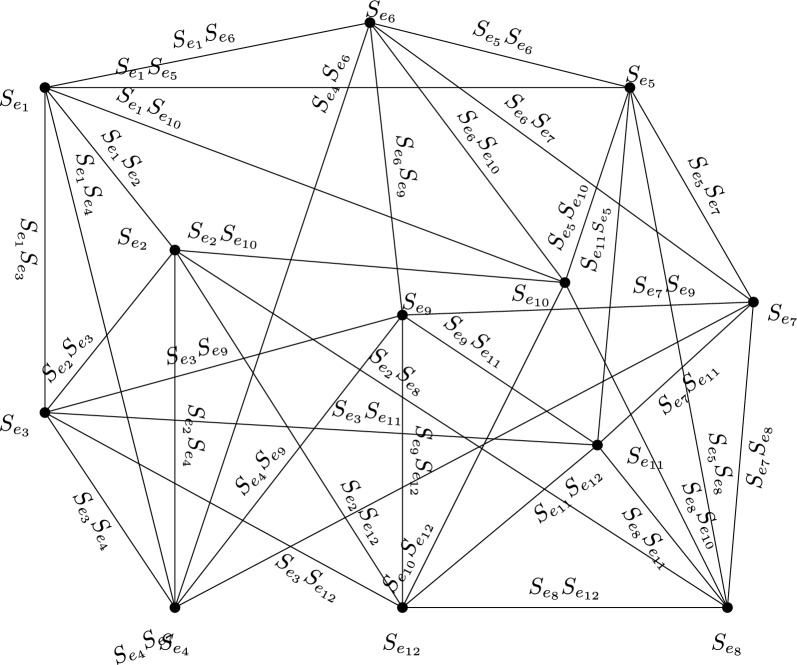
L(H) of an IVBFG H

Consider an IVBFG H from the following Fig. 5. It is $(k_1, k_2)$-regular IVBFG where $(k_1,k_2)=([0.40,0.53],[-0.93,-0.40])$. But, its corresponding line graph shown in Fig. 6 is not regular graph.

The vertex membership values of IVBFLG H.

$$\begin{aligned}{} & {} [ \lambda ^l_{A_L}(S_{e_1}), ~\lambda ^u_{A_L}(S_{e_1})], [\mu ^l_{A_L}(S_{e_1}), ~\mu ^u_{A_L}(S_{e_1})] =([0.09, 0.10 ], [-0.20, -0.10]),\\{} & {} \quad [ \lambda ^l_{A_L}(S_{e_2}), ~\lambda ^u_{A_L}(S_{e_2})], [\mu ^l_{A_L}(S_{e_2}), ~\mu ^u_{A_L}(S_{e_2})] =( [0.09,0.12], [-0.30,-0.10]),\\{} & {} \quad [ \lambda ^l_{A_L}(S_{e_3}), ~\lambda ^u_{A_L}(S_{e_3})], [\mu ^l_{A_L}(S_{e_3}), ~\mu ^u_{A_L}(S_{e_3})] =([0.12,0.16], [-0.23,~-0.1]),\\{} & {} \quad [ \lambda ^l_{A_L}(S_{e_4}), ~\lambda ^u_{A_L}(S_{e_4})], [\mu ^l_{A_L}(S_{e_4}), ~\mu ^u_{A_L}(S_{e_4})]=([0.10,0.15],[-0.20,~-0.10]),\\{} & {} \quad [ \lambda ^l_{A_L}(S_{e_5}), ~\lambda ^u_{A_L}(S_{e_5})], [\mu ^l_{A_L}(S_{e_5}), ~\mu ^u_{A_L}(S_{e_5})]=([0.10,0.15],[-0.20,~-0.10]),\\{} & {} \quad [ \lambda ^l_{A_L}(S_{e_6}), ~\lambda ^u_{A_L}(S_{e_6})], [\mu ^l_{A_L}(S_{e_6}), ~\mu ^u_{A_L}(S_{e_6})]=([0.09,0.12],[-0.30,-0.10]),\\{} & {} \quad [ \lambda ^l_{A_L}(S_{e_7}), ~\lambda ^u_{A_L}(S_{e_7})], [\mu ^l_{A_L}(S_{e_7}), ~\mu ^u_{A_L}(S_{e_7})]=([0.12,0.16], [-0.23,~-0.10]),\\{} & {} \quad [ \lambda ^l_{A_L}(S_{e_8}), ~\lambda ^u_{A_L}(S_{e_8})], [\mu ^l_{A_L}(S_{e_8}), ~\mu ^u_{A_L}(S_{e_8})]=( [0.09,0.10],[-0.20,~-0.10] ),\\{} & {} \quad [ \lambda ^l_{A_L}(S_{e_9}), ~\lambda ^u_{A_L}(S_{e_9})], [\mu ^l_{A_L}(S_{e_9}), ~\mu ^u_{A_L}(S_{e_9})]=( [0.09,0.10],[-0.20,~-0.10] ),\\{} & {} \quad [ \lambda ^l_{A_L}(S_{e_{10}}), ~\lambda ^u_{A_L}(S_{e_{10}})], [\mu ^l_{A_L}(S_{e_{10}}), ~\mu ^u_{A_L}(S_{e_{10}})]=( [0.12,0.16],[-0.23,~-0.1] ),\\{} & {} \quad [ \lambda ^l_{A_L}(S_{e_{11}}), ~\lambda ^u_{A_L}(S_{e_{11}})], [\mu ^l_{A_L}(S_{e_{11}}), ~\mu ^u_{A_L}(S_{e_{11}})]=([0.09,0.12], [-0.30,-0.10]),\\{} & {} \quad [ \lambda ^l_{A_L}(S_{e_{12}}), ~\lambda ^u_{A_L}(S_{e_{12}})], [\mu ^l_{A_L}(S_{e_{12}}), ~\mu ^u_{A_L}(S_{e_{12}})]=([0.10,0.15], [-0.20,~-0.10]). \end{aligned}$$

**Table **1 indicates the edge membership values of line graph of H.

**Table 1:** An edge membership values of IVBFLG H

	$[\lambda ^l_A, ~\lambda ^u_B]$	$[\mu ^l_B, ~\mu ^u_B]$		$[\lambda ^l_A, ~\lambda ^u_B]$	$[\mu ^l_B, ~\mu ^u_B]$
$S_{e_1}S_{e_2}$	[0.09 0.10 ]	[− 0.20 − 0.10]	$S_{e_4}S_{e_{9}}$	[0.09, 0.10]	[− 0.20, − 0.10]
$S_{e_1}S_{e_3}$	[0.09, 0.10]	[− 0.20, − 0.10]	$S_{e_5}S_{e_6}$	[0.09, 0.12]	[− 0.20, − 0.10]
$S_{e_1}S_{e_4}$	[0.09, 0.10]	[− 0.20, − 0.10]	$S_{e_5}S_{e_7}$	[0.10, 0.15]	[− 0.20, − 0.10]
$S_{e_1}S_{e_5}$	[0.09, 0.10]	[− 0.20, − 0.10]	$S_{e_5}S_{e_8}$	[0.09, 0.10]	[− 0.20, − 0.10]
$S_{e_1}S_{e_6}$	[0.09, 0.10]	[− 0.20, − 0.10]	$S_{e_5}S_{e_{10}}$	[0.10, 0.15]	[− 0.20, − 0.1]
$S_{e_1}S_{e_{10}}$	[0.09, 0.10]	[− 0.20,− 0.10]	$S_{e_6}S_{e_{7}}$	[0.09, 0.12]	[− 0.23, − 0.10]
$S_{e_2}S_{e_3}$	[0.09, 0.12]	[− 0.23, − 0.10]	$S_{e_6}S_{e_{9}}$	[0.09, 0.12]	[− 0.30, − 0.10]
$S_{e_2}S_{e_4}$	[0.09, 0.12]	[− 0.20, − 0.10]	$S_{e_6}S_{e_{10}}$	[0.09, 0.10]	[− 0.20, − 0.10]
$S_{e_2}S_{e_8}$	[0.09, 0.10]	[− 0.20, − 0.10]	$S_{e_7}S_{e_{8}}$	[0.09, 0.10]	[− 0.20, − 0.10]
$S_{e_2}S_{e_{10}}$	[0.09, 0.12]	[− 0.23, − 0.1]	$S_{e_7}S_{e_{9}}$	[0.09, 0.10]	[− 0.20, − 0.10]
$S_{e_2}S_{e_{12}}$	[0.09, 0.12]	[− 0.23,− 0.10]	$S_{e_7}S_{e_{11}}$	[0.09, 0.12]	[− 0.23, − 0.10]
$S_{e_3}S_{e_4}$	[0.10, 0.15 ]	[− 0.20 − 0.10]	$S_{e_8}S_{e_{11}}$	[0.09, 0.10]	[− 0.20, − 0.10]
$S_{e_3}S_{e_9}$	[0.09, 0.10 ]	[− 0.20 − 0.10]	$S_{e_8}S_{e_{12}}$	[0.09, 0.10]	[− 0.20, − 0.10]
$S_{e_3}S_{e_{11}}$	[0.09, 0.12]	[− 0.23,− 0.10]	$S_{e_9}S_{e_{11}}$	[0.09, 0.10]	[− 0.20, − 0.10]
$S_{e_3}S_{e_{12}}$	[0.09, 0.15]	[− 0.20, − 0.10]	$S_{e_9}S_{e_{12}}$	[0.09, 0.10]	[− 0.20, − 0.10]
$S_{e_4}S_{e_6}$	[0.09, 0.12]	[− 0.20, − 0.10]	$S_{e_{10}}S_{e_{12}}$	[0.10, 0.15]	[− 0.20, − 0.10]
$S_{e_4}S_{e_7}$	[0.10, 0.15]	[− 0.20, − 0.10]	$S_{e_{11}}S_{e_{12}}$	[0.09, 0.12]	[− 0.20, − 0.10]

### Definition 36

The size of a $k-$regular IVBFG G is $\frac{kn}{2}$; where $|V| =n$ and $k=[k_{1},k_2]$. i.e,

$$\begin{aligned} S(G)=\frac{kn}{2}. \end{aligned}$$

### Definition 37

Let $L(G)= (A_L, B_L)$ is an IVBFLG of graph G. Then *L*(*G*) is called strongly regular IVBFLG if the following conditions are satisfied:-

i) L(G) is a k-regular IVBFLG,
ii) The sum of the membership values of the vertices common to the adjacent vertices in L(G) is the same for all adjacent pairs of vertices,
iii) The sum of the membership values of the vertices common to the non-adjacent vertices in L(G) is the same for all non-adjacent pairs of vertices.

### Proposition 38

Every line graph of an interval valued bipolar graph is strong graph.

### Proof

The proof of this proposition is straightforward from definition of strong graph. $\square$

### Example 39

Consider an IVBFG $G=(A,B)$ where A be a bipolar fuzzy subset of V and B be a bipolar fuzzy subset of E such that $V=<v_1,~v_2, ~v_3>, E=<v_1v_2,~v_2v_3$. Let $\lambda$ be a positive membership value and $\mu$ be a negative membership value of G, defined by

$$\begin{aligned}{} & {} [\lambda _A^l(v_1),\lambda _A^u(v_1) ]= [0.6,0.9],\\{} & {} \quad [\mu _A^l(v_1),\mu _A^u(v_1) ]=[-0.7,~-0.3]\\{} & {} \quad [\lambda _A^l(v_2),\lambda _A^u(v_2) ]= [0.3,0.7],\\{} & {} \quad [\mu _A^l(v_2),\mu _A^u(v_2) ]=[-0.6,~-0.4]\\{} & {} \quad [\lambda _A^l(v_3),\lambda _A^u(v_3) ]= [0.4,0.6],\\{} & {} \quad [\mu _A^l(v_3),\mu _A^u(v_3) ]=[-0.8,~-0.2] and\\{} & {} \quad [\lambda _B^l(e_1),\lambda _B^u(e_1) ]= [0.3,0.6],\\{} & {} \quad [\mu _B^l(e_1),\mu _B^u(e_1) ]=[-0.6,~-0.2]\\{} & {} \quad [\lambda _B^l(e_2),\lambda _B^u(e_2) ]= [0.3,0.7], [\mu _B^l(e_2),\mu _B^u(e_2) ]=[-0.6,~-0.3]. \end{aligned}$$

By routine computations the line graph of IVBFG G is strong.

### Proposition 40

The IVBFLG L(G) is connected iff its original graph IVBFG G is connected graph.

### Proof

Given G is a IVBFG and *L*(*G*) is connected interval valued bipolar fuzzy line graph of G. First, we must demonstrate that precondition. Assume G is disconnected IVBFG. Then G has at least two nodes that are not connected by a path. If we choose one edge from the first component, there are no edges that are adjacent to edges in other components of G. The *L*(*G*) of G is then broken and contradicting. So that, G must be connected. Conversely, assume that G is connected IVBFG. We need to show that *L*(*G*) is connected. Since G is connected, there is a path that connects each pair of nodes. Adjacent edges in G are thus neighboring nodes in *L*(*G*) , according to the definition of *L*(*G*) . As a result, each pair of nodes in L(G) has a path that connects them. The proof completed. $\square$

### Proposition 41

An IVBFLG is always a strong IVBFG.

### Proof

It is straightforward from the definition, therefore it is omitted. $\square$

### Proposition 42

Let $G = (A_1, B_1)$ be an interval-valued bipolar fuzzy graph of $G^*$ and $P(G) = (A_2, B_2)$ be an interval-valued bipolar fuzzy intersection graph of *P*(*S*). Then,

- an interval-valued bipolar fuzzy intersection graph is an interval-valued bipolar fuzzy graph.
- an interval-valued bipolar fuzzy graph is isomorphic to an interval-valued bipolar fuzzy intersection graph

### Proof

- From Definition 22, it follows that
  $$\begin{aligned} \lambda _{B_2}(S_iS_j)&=[\lambda _{B_2}^l(S_iS_j),~\lambda _{B_2}^u(S_iS_j)]\\&=[\lambda _{B_1}^l(v_iv_j),~\lambda _{B_1}^u(v_iv_j)]\\&\le [\min (\lambda _{A_1}^l(v_i), \lambda _{A_1}^l(v_j)),~\min (\lambda _{A_1}^u(v_i), \lambda _{A_1}^u(v_j))]\\ \end{aligned}$$
  $$\begin{aligned} \mu _{B_2}(S_iS_j)&=[\mu _{B_2}^l(S_iS_j),~\mu _{B_2}^u(S_iS_j)]\\&=[\mu _{B_1}^l(v_iv_j),~\mu _{B_1}^u(v_iv_j)]\\&\ge [\max (\mu _{A_1}^l(v_i), \mu _{A_1}^l(v_j)),~\max (\mu _{A_1}^u(v_i), \mu _{A_1}^u(v_j))]\\ \end{aligned}$$
  This implies that an interval-valued bipolar fuzzy intersection graph is an interval-valued bipolar fuzzy graph.
- Define $\varphi : V \rightarrow S$ by $\varphi (v_i) = s_i,$ for $i = 1, 2,\cdots , n.$ Clearly, $\varphi$ is a one-to-one function of V onto S. Now $vivj\in E$ if and only if $s_is_j\in T$ and $T = {\varphi (v_i)\varphi (v_j ):~ v_iv_j\in E}.$ Also
  $$\begin{aligned} \lambda _{A_2}(\varphi (v_i))&=[\lambda _{A_2}^l(\varphi (v_i)), \lambda _{A_2}^u(\varphi (v_i))]\\&=[\lambda _{A_2}^l(S_i), \lambda _{A_2}^u(S_i)]\\&=[\lambda _{A_1}^l(v_i), \lambda _{A_1}^u(v_i)]\\ \\ \mu _{A_2}(\varphi (v_i))&=[\mu _{A_2}^l(\varphi (v_i)), \mu _{A_2}^u(\varphi (v_i))]\\&=[\mu _{A_2}^l(S_i), \mu _{A_2}^u(S_i)]\\&=[\mu _{A_1}^l(v_i), \mu _{A_1}^u(v_i)]\\ \\ \lambda _{B_2}(\varphi (v_i)\varphi (v_j))&=[\lambda _{B_2}^l(\varphi (v_i)\varphi (v_j)), \lambda _{A_B}^u(\varphi (v_i)\varphi (v_j))]\\&=[\lambda _{B_2}^l(S_iS_j), \lambda _{B_2}^u(S_iS_j)]\\&=[\lambda _{B_2}^l(v_iv_j), \lambda _{B_2}^u(v_iv_j)]\\ \\ \mu _{B_2}(\varphi (v_i)\varphi (v_j))&=[\mu _{B_2}^l(\varphi (v_i)\varphi (v_j)), \mu _{A_B}^u(\varphi (v_i)\varphi (v_j))]\\&=[\mu _{B_2}^l(S_iS_j), \mu _{B_2}^u(S_iS_j)]\\&=[\mu _{B_2}^l(v_iv_j), \mu _{B_2}^u(v_iv_j)]\\ \end{aligned}$$
  Thus $\varphi$ is an isomorphism of *G* onto *P*(*G*) .

$$\square$$

### Proposition 43

Let $G_1$ and $G_2$ IVBFGs of $G_1^*$ and $G_2^*$ respectively. If the mapping $\psi : G_1 \rightarrow G_2$ is a weak isomorphism, then $\psi : G_1^*\rightarrow G_2^*$ is an isomorphism map.

### Proof

Suppose $\psi : G_1 \rightarrow G_2$ is a weak isomorphism. Then

$$\begin{aligned}{} & {} \text {u}\in V_1 \Leftrightarrow \psi (\text {u})\in V_2 ~~\textrm{and} \\{} & {} \quad \text {u}\text {v}\in E_1 \Leftrightarrow \psi (\text {u})\psi (\text {v})\in E_2. ~~ \end{aligned}$$

Hence the proof. $\square$

### Theorem 44

Given a IVBFLG $L(G) = (A_L, B_L)$ corresponding to IVBFG $G = (A, B)$. If the crisp graph $G^*= (V, E)$ corresponding to G is connected, then

1. There exists a map $\psi :G\rightarrow L(G)$ which is a weak isomorphism iff $G^*$ a cycle graph and, $A = (\lambda _{A}, \mu _{A})$ and $B = (\lambda _{B}, \mu _{B})$ are constant functions. i.e., $\lambda _{A}(\text {u}) =\lambda _{B}(e)\Rightarrow [\lambda _{A}^l(\text {u}), \lambda _{A}^u(\text {u})]=[ \lambda _{B}^l(e), \lambda _{B}^u(e) ],$ and $\mu _{A}(\text {u}) =\mu _{B}(\text {u}) \Rightarrow [ \mu _{A}^l(\text {u}), \mu _{A}^u(\text {u})]=[ \mu _{B}^l(e), \mu _{B}^u(e) ]$, $\forall \text {u} \in V, e\in E,$ where $A= ([\lambda _{A}^l,\lambda _{A}^u],[\mu _{A}^l,\mu _{A}^u])$ and $B= ([\lambda _{B}^l,\lambda _{B}^u],[\mu _{B}^l,\mu _{B}^u])$.
2. If a map $\psi :G\rightarrow L(G)$ is a weak isomorphism then $\psi$ is isomorphism.

### Proof

Lets consider a weak isomorphism map $\psi :G\rightarrow L(G)$ is exists. Then its a weak vertex and a weak line isomorphism. Then we have

- $[ \lambda _{ A}^l (\text {u}_i),\lambda _{ A}^u (\text {u}_i)]= [\lambda _{ A_L}^l (\psi (\text {u}_i)),\lambda _{ A_L}^u (\psi (\text {u}_i))]$, $[ \mu _{ B}^l (\text {u}_i), \mu _{ B}^u (\text {u}_i)]= [ \mu _{ B_L}^l (\psi (\text {u}_i)), \mu _{ B_L}^u (\psi (\text {u}_i))]$, for every vertex $\text {u}_i\in V.$
- $$[\lambda _{B}^l (\text {u}_i\text {u}_j),~ \lambda _{B}^u(\text {u}_i\text {u}_j)]= [\lambda _{B_L}^l( \psi (\text {u}_i) \psi (\text {u}_j)), ~\lambda _{B_L}^u( \psi (\text {u}_i) \psi (\text {u}_j))]$$ $$\mu _{B} (\text {u}_i\text {u}_j)=[ \mu _{B}^l (\text {u}_i\text {u}_j), \mu _{B}^u(\text {u}_i\text {u}_j)]= [ \mu _{B_L}^l( \psi (\text {u}_i) \psi (\text {u}_j)), ~ \mu _{B_L}^u( \psi (\text {u}_i) \psi (\text {u}_j))],~ \forall \text {u}_i\text {u}_j\in E.$$

This means that a crisp graph $G^*= (V, E)$ is a cycle graph from proposition 43.

Now, assume that the $V=\{\text {u}_1, \text {u}_2,\cdots , \text {u}_n\}$, $E=\{e_1=\text {u}_1\text {u}_2,~e_2= \text {u}_2\text {u}_3,\cdots , e_n=\text {u}_n\text {u}_1\}$ and $C=\text {u}_1\text {u}_2\text {u}_3 \cdots \text {u}_n\text {u}_1$ is a cycle of $G^*$. Then we have IVBFS

$$[\lambda _{A}^l(\text {u}_i), \lambda _{A}^u(\text {u}_i)]=[t_i^l, ~t_i^u]$$, $$[\mu _{A}^l(\text {u}_i), \mu _{A}^u(\text {u}_i)]=[f_i^l, ~f_i^u]$$

$$\lambda _{B}(\text {u}_i\text {u}_{i+1})=[\lambda _{B}^l(\text {u}_i\text {u}_{i+1}),~\lambda _{B}^u(\text {u}_i\text {u}_{i+1})]=[r_i^l,~ r_i^u]$$

$\mu _{B}(\text {u}_i\text {u}_{i+1})=[\mu _{B}^l(\text {u}_i\text {u}_{i+1}),~\mu _{B}^u(\text {u}_i\text {u}_{i+1})]=[q_i^l,~ q_i^u],$ where $i=1, 2, \cdots , n ~ \textrm{and}~ \text {u}_{n+1}=\text {u}_1.$ Thus, for $t_1^l= t_{n+1}^l, t_1^u= t_{n+1}^u, ~f_1^l= f_{n+1}^l, f_1^u= f_{n+1}^l$ we know that

1 $$\begin{aligned}&r_i^l\le \min (t_i^l, ~t_{i+1}^l), r_i^u\le \min ( t_i^u, ~t_{i+1}^u)\\&q_i^l\ge \max (f_i^l, ~ f_{i+1}^l), q_i^u\ge \max (f_i^u,~f_{i+1}^u). \end{aligned}$$

Now, we have a line graph of $L(G^*)=(Z, W)$ where $Z=\{ S_{e_i} \} ~\textrm{and }~~W=\{ S_{e_i}S_{e_{i+1}} \}.$ And also,

$$\begin{aligned}{}[\lambda _{A_L}^l(S_{e_i}),\lambda _{A_L}^u(S_{e_i}) ]&=[\lambda _{B}^l(e_i), \lambda _{B}^u(e_i)]\\&=[\lambda _{B}^l(\text {u}_i\text {u}_{i+1}), \lambda _{B}^u(\text {u}_i\text {u}_{i+1})]\\&=[r^l_i , r^u_i] \\ [\mu _{A_L}^l(S_{e_i}),\mu _{A_L}^u(S_{e_i}) ]&=[\mu _{B}^l(e_i), \mu _{B}^u(e_i)]\\&=[\mu _{B}^l(\text {u}_i\text {u}_{i+1}), \mu _{B}^u(\text {u}_i\text {u}_{i+1})]\\&=[q^l_i , q^u_i] \\ \lambda _{B_L}^u(S_{e_i}S_{e_{i+1}})&=min \{ \lambda _{B}^u(e_i),\lambda _{B}^u(e_{i+1})\}\\ {}&=min\{\lambda _{B}^u(\text {u}_i\text {u}_{i+1}), \lambda _{B}^u(\text {u}_{i+1}\text {u}_{i+2}) \}\\&= min\{r^u_i,r^u_{i+1}\} \\ \lambda _{B_L}^l(S_{e_i}S_{e_{i+1}})&=min \{ ~\lambda _{B}^l(e_i),\lambda _{B}^l(e_{i+1})\}\\ {}&=min\{~\lambda _{B}^l(\text {u}_i\text {u}_{i+1}), \lambda _{B}^l(\text {u}_{i+1}\text {u}_{i+2}) \}\\&= ~min\{r^l_i,r^l_{i+1}\} \\ \mu _{B_L}^u(S_{e_i}S_{e_{i+1}})&=max \{ \mu _{B}^u(e_i),\mu _{B}^u(e_{i+1})\}\\ {}&=max\{\mu _{B}^u(\text {u}_i\text {u}_{i+1}), \mu _{B}^u(\text {u}_{i+1}\text {u}_{i+2}) \}\\&= max\{q^u_i,q^u_{i+1}\} \\ \mu _{B_L}^l(S_{e_i}S_{e_{i+1}})&=max \{ \mu _{B}^l(e_i),\mu _{B}^l(e_{i+1})\}\\ {}&=max\{\mu _{B}^l(\text {u}_i\text {u}_{i+1}), \mu _{B}^l(\text {u}_{i+1}\text {u}_{i+2}) \}\\&= max\{q^l_i,q^l_{i+1}\} \end{aligned}$$

where $\text {u}_{n+1}=\text {u}_1,~\text {u}_{n+2}=\text {u}_2$, $r^u_1=r^u_{n+1}, r^l_1=r^l_{n+1},~ q^u_{n+1} =r^u_1,$, $q^l_{n+1} =q^l_1$, and $i=1, 2, \cdots , n$. Then $\psi : V\rightarrow H$ is bijective map since $\psi : G^*\rightarrow L(G^*)$ is isomorphism. And also, $\psi$ preserves adjacency. So that $\psi$ induces a permutation $\pi$ of $\{1, 2, \cdots , n \}$ which $\psi (\text {u}_i)=S_{e_{\pi (i)} }$

and for $e_i=\text {u}_i\text {u}_{i+1}$ then $\psi (\text {u}_i)\psi (\text {u}_{i+1})=S_{e_{\pi (i)}} S_{e_{\pi (i+1)}}, ~i=1,2,\cdots ,n-1$.

Now

$$t_i^l=\lambda _{A}^l(\text {u}_i)\le \lambda _{A_L}^l(\psi (\text {u}_i))= \lambda _{A_L}^l(S_{e_{\pi (i)} }) =r_{\pi (i)}^l$$,

$$t_i^u=\lambda _{A}^u(\text {u}_i)\le \lambda _{A_L}^u(\psi (\text {u}_i))= \lambda _{A_L}^u(S_{e_{\pi (i)} }) =r_{\pi (i)}^u$$,

$$f_i^l=\mu _{A}^l(\text {u}_i)\ge \mu _{A_L}^l(\psi (\text {u}_i))= \mu _{A_L}^l(S_{e_{\pi (i)} })=q_{\pi (i)} ^l$$,

$$f_i^u=\mu _{A}^u(\text {u}_i)\ge \mu _{A_L}^u(\psi (\text {u}_i))= \mu _{A_L}^u(S_{e_{\pi (i)} })=q_{\pi (i)} ^u$$.

Hence,

2 $$\begin{aligned}&t^l_i\le r^l_{\pi (i)}, ~~~~t_i^u\le ~ r_{\pi (i)}^u \\&f^l_i\le q^l_{\pi (i)}, \,~~~~ f_i^u\le q^u_{\pi (i)} \end{aligned}$$

And for $$e_i=\text {u}_i\text {u}_{i+1}$$,

3 $$\begin{aligned} r_i^l = \lambda _{B}^l(\text {u}_i\text {u}_{i+1})&\le \lambda _{B_L}^l(\psi (\text {u}_i)\psi (\text {u}_{i+1}) \nonumber \\&=\lambda _{B_L}^l(S_{e_{\pi (i)} } S_{e_{\pi (i+1} })) \nonumber \\&=min\{\lambda _{B}^l(e_{\pi (i)}) , \lambda _{B}^l( e_{\pi (i+1)} )\} \nonumber \\&=min\{r_{\pi (i)}^l, r_{\pi (i+1)}^l\} \nonumber \\ r_i^u = \lambda _{B}^u(\text {u}_i\text {u}_{i+1}))&\le \lambda _{B_L}^u(\psi (\text {u}_i)\psi (\text {u}_{i+1}) \nonumber \\&=\lambda _{B_L}^u(S_{e_{\pi (i)} } S_{e_{\pi (i+1} })) \nonumber \\&=min\{\lambda _{B}^u(e_{\pi (i)}) , \lambda _{B}^u( e_{\pi (i+1)} )\} \nonumber \\&=min\{r_{\pi (i)}^u, r_{\pi (i+1)}^u\} \nonumber \\ q_i^l = \mu _{B}^l(\text {u}_i\text {u}_{i+1})&\ge \mu _{B_L}^l(\psi (\text {u}_i)\psi (\text {u}_{i+1}) \nonumber \\&=\mu _{B_L}^l(S_{e_{\pi (i)} } S_{e_{\pi (i+1} })) \nonumber \\&=max\{\mu _{B}^l(e_{\pi (i)}) , \mu _{B}^l( e_{\pi (i+1)} )\} \nonumber \\&=max\{q_{\pi (i)}^l, q_{\pi (i+1)}^l\} \nonumber \\ q_i^u = \mu _{B}^u(\text {u}_i\text {u}_{i+1})&\ge \mu _{B_L}^u(\psi (\text {u}_i)\psi (\text {u}_{i+1}) \nonumber \\&=\mu _{B_L}^u(S_{e_{\pi (i)} } S_{e_{\pi (i+1} })) \nonumber \\&=max\{\mu _{B}^u(e_{\pi (i)}) , \mu _{B}^u( e_{\pi (i+1)} )\} \nonumber \\&=max\{q_{\pi (i)}^u, q_{\pi (i+1)}^u\} ~~for ~i=1,2,\cdots , n .\nonumber \\&r^l_i\le min\{ r^l_{\pi (i)}, r^l_{\pi (i+1)}\} , \, ~~~~r^u_i\le min\{ r^u_{\pi (i)}, r^u_{\pi (i+1)}\} \nonumber \\&q^l_i\ge max\{ q^l_{\pi (i)}, q^l_{\pi (i+1)}\} , \, ~~~~q^u_i\ge max\{ q^u_{\pi (i)}, q^u_{\pi (i+1)}\}. \end{aligned}$$

Thus from Eq. 3, we get $r_{i}^l\le r_{\pi (i)}^l, ~ r^u_i\le r^u_{\pi (i)},~ q^l_i\ge q^l_{\pi (i)}$ and $q^u_i\ge q^u_{\pi (i)}$. and also $r^l_{\pi (i)} \le r^l_{\pi (\pi (i))}, ~ r^u_{\pi (i)} \le r^u_{\pi (\pi (i))},~ q^l_{\pi (i)} \ge q^l_{\pi (\pi (i))}$and $q^u_{\pi (i)} \ge q^u_{\pi (\pi (i))}$. By proceeding this process, we get

$$\begin{aligned}{} & {} r^l_i \le r^l_{\pi (i)}\le \cdots \le r^l_{\pi ^k(i)} \le r_i^l \\{} & {} \quad r^u_i \le r^u_{\pi (i)}\le \cdots \le r^u_{\pi ^k(i)} \le r_i^u \\{} & {} \quad q^l_i \le q^l_{\pi (i)}\ge \cdots \ge q^l_{\pi ^k(i)} \ge q_i^l \\{} & {} \quad q^u_i \ge q^u_{\pi (i)}\ge \cdots \ge q^u_{\pi ^k(i)} \ge q_i^u \end{aligned}$$

where $\pi ^{k+1}$ is the identity function. It follows $r^l_{\pi (i)} = r^l_{\pi (\pi (i))}, ~ r^u_{\pi (i)} = r^u_{\pi (\pi (i))},~ q^l_{\pi (i)} = q^l_{\pi (\pi (i))}$ and $q^u_{\pi (i)} = q^u_{\pi (\pi (i))}$. Again, from Eq. 3, we get

$$r^l_{i}\le r^l_{\pi (i+1)}=r^l_{i+1},~~ r^u_{i}\le r^u_{\pi (i+1)} =r^u_{i+1}$$

$$q^l_{i}\ge q^l_{\pi (i+1)} =q^l_{i+1}, q^u_{i}\ge q^u_{\pi (i+1)} =q^l_{i+1}.$$

This implies for all $i=1,2,\cdots ,n$, $r^l_{i}=r^l_{1}, r^u_{i}=r^u_{1}, q^l_{i}=q^l_{1}$ and $q^u_{i}=q+_{1}$. Thus, from Eq. 1 and 2 we obtain

$$\begin{aligned}{} & {} r^l_1=~\cdots ~=r^l_n=t^l_1=\cdots =t^l_n \\{} & {} \quad r^u_1=\cdots =r^u_n~=~t^u_1=\cdots =t^u_n \\{} & {} \quad q^l_1=\cdots =q^l_n~=~f^l_1=\cdots =f^l_n \\{} & {} \quad q^u_1=\cdots =q^u_n ~=f^u_1=\cdots =f^u_n~. \end{aligned}$$

Finally, the proof of the second part is forwarded from part one. That is, if $\psi :G\rightarrow L(G)$ is a weak isomorphism then a mapping $\psi$ is isomorphism map. $\square$

### Proposition 45

An IVBFLG is the generalization of the interval valued fuzzy line graph.

### Proof

Let $L(G) = (A_L, B_L)$ be an IVBFLG. Then, by setting both lower and upper negative upper-membership values of nodes and edges to zero, which reduces an interval valued bipolar fuzzy line graph to interval valued fuzzy line graph. $\square$

## Limitations

This paper was presented the concept of IVBFLG and some of its mathematical properties developed. Moreover, some remarkable properties of such as strong IVBFLG, regularity of IVBFLG and complete IVBFLG have been investigated and illustrated with the examples. Based on these ideas, we can apply IVBFG to other graph theory areas, as well as build a network model for IVBFG and develop an algorithm-oriented solution. We also give a necessary and sufficient condition for a IVBFG to be isomorphic to its corresponding IVBFLG. However, the researchers considered only undirected simple IVBFG and the applications of this proposed graph are not included in this paper. So that, in the future work we will apply the concept of IVBFLG on real-life problem and extend to soft fuzzy graph and neutrosophic graph.

## Data Availability

Not applicable.
